# Guanylate Binding Proteins Restrict Leishmania donovani Growth in Nonphagocytic Cells Independent of Parasitophorous Vacuolar Targeting

**DOI:** 10.1128/mBio.01464-20

**Published:** 2020-07-28

**Authors:** Arun Kumar Haldar, Utsav Nigam, Masahiro Yamamoto, Jörn Coers, Neena Goyal

**Affiliations:** a Division of Biochemistry, Central Drug Research Institute, Council of Scientific and Industrial Research, Lucknow, India; b Department of Immunoparasitology, Research Institute for Microbial Diseases, Osaka University, Osaka, Japan; c Department of Molecular Genetics and Microbiology, Duke University Medical Center, Durham, North Carolina, USA; d Department of Immunology, Duke University Medical Center, Durham, North Carolina, USA; UC Berkeley

**Keywords:** *Leishmania*, nonphagocytic cells, guanylate binding proteins, GBP, GTPases, IRGM, autophagy, Atg3, LAMP, LC3, cell-autonomous immunity, interferons

## Abstract

Interferon (IFN)-inducible guanylate binding proteins (GBPs) play important roles in host defense against many intracellular pathogens that reside within pathogen-containing vacuoles (PVs). For instance, members of the GBP family translocate to PVs occupied by the protozoan pathogen *Toxoplasma* and facilitate PV disruption and lytic parasite killing. While the GBP defense program targeting *Toxoplasma* has been studied in some detail, the role of GBPs in host defense to other protozoan pathogens is poorly characterized. Here, we report a critical role for both mouse and human GBPs in the cell-autonomous immune response against the vector-borne parasite Leishmania donovani. Although L. donovani can infect both phagocytic and nonphagocytic cells, it predominantly replicates inside professional phagocytes. The underlying basis for this cell type tropism is unclear. Here, we demonstrate that GBPs restrict growth of L. donovani in both mouse and human nonphagocytic cells. GBP-mediated restriction of L. donovani replication occurs via a noncanonical pathway that operates independent of detectable translocation of GBPs to L. donovan-containing vacuoles (LCVs). Instead of promoting the lytic destruction of PVs, as reported for GBP-mediated killing of *Toxoplasma* in phagocytic cells, GBPs facilitate the delivery of L. donovani into autolysosomal-marker-positive compartments in mouse embryonic fibroblasts as well as the human epithelial cell line A549. Together our results show that GBPs control a novel cell-autonomous host defense program, which renders nonphagocytic cells nonpermissible for efficient *Leishmania* replication.

## Abstract (precis)

The obligate intracellular parasite *Leishmania* causes the disease leishmaniasis, which is transmitted to mammalian hosts, including humans, via the sandfly vector. Following the bite-induced breach of the skin barrier, *Leishmania* is known to live and replicate predominantly inside professional phagocytes. Although *Leishmania* is also able to infect nonphagocytic cells, nonphagocytic cells support limited parasitic replication for unknown reasons. In this study, we show that nonphagocytic cells possess an intrinsic property to restrict *Leishmania* growth. Our study defines a novel role for a family of host defense proteins, the guanylate binding proteins (GBPs), in antileishmanial immunity. Mechanistically, our data indicate that GBPs facilitate the delivery of *Leishmania* into antimicrobial autolysosomes, thereby enhancing parasite clearance in nonphagocytic cells. We propose that this GBP-dependent host defense program makes nonphagocytic cells an inhospitable host cell type for *Leishmania* growth.

## INTRODUCTION

Interferon (IFN)-inducible GTPases guanylate binding proteins (GBPs) are key factors in cell-autonomous immunity and play an important role in inflammasome-driven responses during intracellular pathogenic infections, inflammation, and cancer (1–6). Currently, 7 human GBPs and 11 mouse GBPs have been identified. The seven human *GBP* genes reside within a single cluster on human chromosome 1. Genes encoding mouse Gbp1, Gbp2, Gbp3, Gbp5, and Gbp7 (Gbp^chr3^ proteins) are clustered on chromosome 3, whereas those genes encoding Gbp4, Gbp6, Gbp8, Gbp9, Gbp10, and Gbp11 are located on chromosome 5 (4, 7, 8). GBPs reside predominately in the cytoplasm, with some association with intracellular membranes, within vesicle-like structures or in the nucleus (9–12). These proteins are highly conserved and belong to the dynamin superfamily of large GTPases (13) which bind GTP and GDP and hydrolyze these nucleotides to GDP and then further to GMP (14–17). Members of the GBP family form homo- and heterodimers (13, 18, 19). Supramolecular GBP complexes can directly attack pathogen-containing vacuoles (PVs) and promote parasite killing (12) and also interfere with virus replication (20–23) or virion assembly (24). Indeed, it is now evident that GBPs play an important role as host resistant factors in response to different infectious pathogens, including viruses, both vacuolar and cytosolic bacteria, and protozoan pathogens (3, 4, 25–27).

*Leishmania* is an obligatory intracellular mammalian pathogen and transmitted by the bite of sandflies. The *Leishmania* infection can remain asymptomatic or result in a wide range of clinical manifestations depending on the species and strains of *Leishmania* and the immunological state of the host. Cutaneous leishmaniasis (CL) manifests as localized skin lesions that may resolve but can become chronic, leading to severe tissue destruction and disfigurement. CL is caused by Leishmania major, Leishmania mexicana, Leishmania amazonensis, and Leishmania tropica. Mucocutaneous leishmaniasis (ML) caused by Leishmania braziliensis is clinically characterized by the involvement of the nasal and oropharyngeal mucosa with extensive tissue destruction due to inflammation. Visceral leishmaniasis (VL), the most severe form of leishmaniasis, is caused by Leishmania donovani and Leishmania infantum. Clinical manifestations of VL include irregular bouts of fever, weight loss, hepatosplenomegaly, the enlargement of liver and spleen, due to parasite infiltration of the liver and spleen, and it is almost always fatal if left untreated (28, 29).

*Leishmania* promastigotes are inoculated into the dermis through the sandfly bite and then phagocytosed by neutrophils. Inside neutrophils, the parasite transforms into the amastigote form (30). The amastigote induces apoptotic host cell death in neutrophils. Parasite-containing apoptotic bodies are then ingested by other professional phagocytes like macrophages and dendritic cells, thereby propagating the infection (31–33). Because amastigotes are mainly observed inside phagocytic cells, like macrophages and dendritic cells, these cells are considered to be the most important host cells in the pathogenesis of leishmaniasis and constitute the best-established infection models. Yet, nonphagocytic cells such as epithelial cells (34) and fibroblasts (35–37) have also been shown to endocytose *Leishmania* and harbor amastigotes in cell culture and *in vivo.* However, interactions between *Leishmania* and nonphagocytic host cells remain poorly characterized.

Although many studies revealed important host protective roles of GBPs in response to infections with different viral, bacterial, and protozoan pathogens, very little is known about possible functions for GBPs in the host defense to *Leishmania* infections. Previous reports showed increased gene expression of Gbp1, Gbp2, Gbp3, Gbp6, and Gbp7 in L. major-infected mouse bone marrow-derived macrophages (BMDMs) (38). It was also shown that Gbp1 and Gbp5 expression was elevated in the skin, lymph nodes, spleen, and liver of L. major-infected mice (39). Dendritic cells, generated from healthy human blood, also exhibited increased expression of GBP1 and GBP2 when infected with L. major, whereas dendritic cells infected by L. donovani had increased expression of only GBP1 (40). Increased human GBP expression was furthermore observed in the skin of patients infected with L. braziliensis (27). Collectively, these studies show that mouse and human *GBP* gene expression is increased in immune cells as well as in skin tissue in response to infections with different *Leishmania* spp. However, potential functions for GBPs as host protective factors against leishmanial infection were not previously explored.

In this study, we demonstrate that mouse Gbps (mGbps) encoded on chromosome 3 (Gbp^chr3^) and human GBP1 (hGBP1) limit *Leishmania* parasite growth in nonphagocytic mouse and human cells. We confirm that L. donovani promastigotes can efficiently infect nonphagocytic mouse embryonic fibroblasts (MEFs) as well as human epithelial A549 cells and transform into its intracellular amastigote form inside these cells. We find that nonphagocytic cells have intrinsic properties to kill the parasites in a gamma interferon (IFN-γ)-independent but GBP-dependent manner. Most previous studies suggested that the recruitment of mGbps and hGBP1 to pathogen-containing vacuoles played an important role in providing cell-autonomous immunity against intravacuolar bacterial and protozoan pathogens. Here, we show that several mouse Gbps (Gbp1, Gbp2, and Gbp5) as well as human GBP1 fail to target *Leishmania*-containing vacuoles (LCVs) but nevertheless restrict parasite burden by facilitating the autolysosomal entrapment of LCVs. Our study thus describes a novel GBP-mediated cell-autonomous defense pathway active against L. donovani in nonphagocytic cells.

## RESULTS

### IFN-γ-independent killing of L. donovani by mouse embryonic fibroblasts.

**FIG 1:**
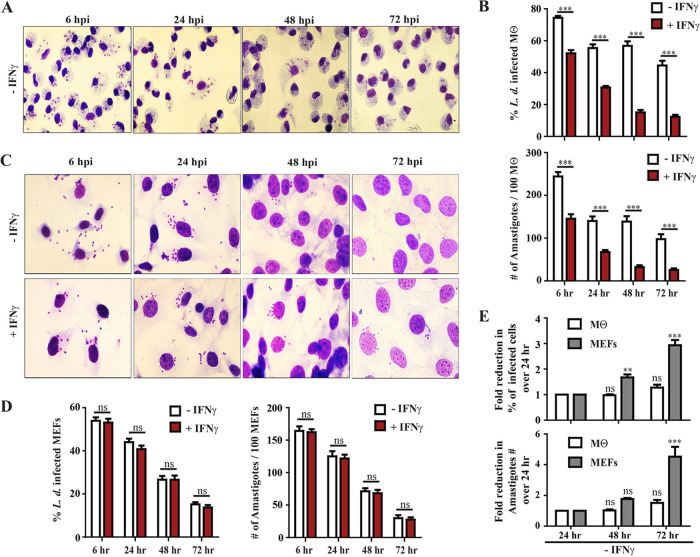
MEFs are efficiently infected by L. donovani, and unlike macrophages, MEFs control parasite growth by an IFN-γ-independent manner. Primary peritoneal macrophages (Mθ) (A and B) and wild-type (WT) MEFs (C and D) were both left unprimed or primed overnight with 200 U/ml of mouse IFN-γ (mIFN-γ) and infected with L. donovani (*L*. *d*.) strain AG83 as described in Materials and Methods. At 6 h postinfection (hpi), floated parasites were washed and incubated further up to the indicated time points. At 6, 24, 48, and 72 hpi, cells were fixed with methanol and stained with Giemsa. (A and C) Representative microscopic images of infected cells at different time points are shown here. (B and D) By using light microscopy, the number of infected cells and the number of intracellular parasites at 6, 24, 48, and 72 hpi were assessed via the quantification of parasite-containing cells and internalized parasites, respectively, as described in Materials and Methods. (E) In unprimed cells, the fold reduction of infected cells over time was calculated as [(percentage of infected cells at 24 hpi/percentage of infected cells at the indicated time postinfection)]. Similarly, fold reduction of amastigote numbers over time was calculated as [(number of intracellular amastigotes per 100 cells at 24 hpi/number of intracellular amastigotes per 100 cells at the indicated time postinfection)]. Results are expressed as means ± standard errors of means (SEM) (error bars) and are based on three independent experiments run in triplicate. Statistical significance was analyzed by two-way ANOVA and indicated as follows: *, *P* < 0.05; **, *P* < 0.01; ***, *P* < 0.001; ns, not significant.

Previous studies showed that phagocytic cells, e.g., macrophages and dendritic cells, are the primary host cell type for *Leishmania* species replication. However, it was also reported that L. major and L. amazonensis actively invade and replicate within fibroblasts (36, 37). To expand on these reported observations, we tested whether L. donovani was able to efficiently invade and infect mouse embryonic fibroblasts (MEFs). We assessed parasite loads in L. donovani-infected primary starch-elicited and adherent peritoneal exudate cells (PECs, henceforth also referred to as macrophages) as well as C57BL/6-derived MEFs. We observed similar infection rates in both PECs and MEFs by assessing burden in Giemsa-stained cells (Fig. 1A to D). We found that greater than 50% of MEFs were infected with L. donovani promastigotes at 2 h postinfection (hpi) (data not shown), and there were no substantial changes up to 6 hpi (Fig. 1C and D). Unexpectedly, these studies revealed that unprimed MEFs showed more efficient leishmanicidal activity compared to unprimed macrophages between 24 hpi to 72 hpi (2.93 ± 0.2-fold versus 1.27 ± 0.11-fold decrease in number of infected cells at 72 hpi over 24 hpi in MEFs versus macrophages and 4.52 ± 0.63-fold versus 1.51 ± 0.19-fold decrease in the number of amastigotes per 100 cells at 72 hpi over 24 hpi in MEFs versus macrophages) (Fig. 1E). There was a significant decrease of the number of infected cells (2.93 ± 0.2-fold decrease at 72 hpi over 24 hpi) as well as the number of amastigotes per 100 cells (4.52 ± 0.63-fold decrease at 72 hpi over 24 hpi) over time in MEFs (Fig. 1E). Some restriction of L. donovani burden was also observed in unprimed macrophages at early stages of the infection which may be linked to the immunostimulatory effect of peritoneal elicitation (41–43). We also observed that the pretreatment of MEFs with 200 U/ml mouse-recombinant gamma interferon (m-IFN-γ) did not have any additional leishmanicidal effect (Fig. 1C and D), whereas it significantly potentiated the anti-*Leishmania* activity of macrophages (Fig. 1B). Confirming the bioactivity of IFN-γ used in our studies, we detected robust induction of mGbp2 by Western blotting in IFN-γ-primed cells (see Fig. S1A in the supplemental material). These data collectively demonstrated that the MEFs possess an intrinsic host defense mechanism to kill intracellular amastigotes in an IFN-γ-independent manner.

**FIG 2:**
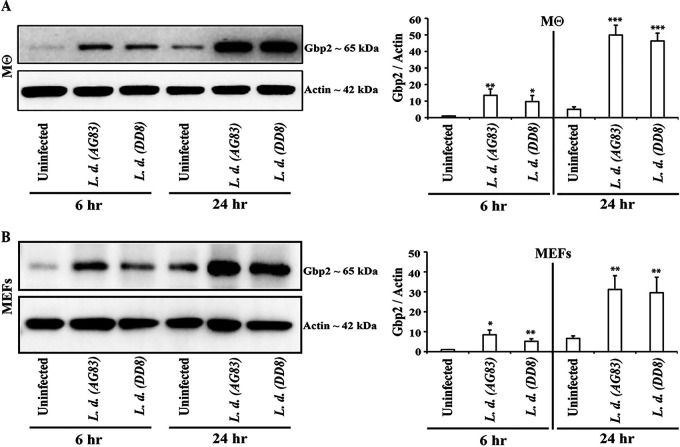
Upregulation of mGbp2 expression in L. donovani-infected macrophages and MEFs. Peritoneal macrophages (A) and MEFs (B), derived from C57BL/6, were either left uninfected or infected with L. donovani strains AG83 and DD8 for 6 h and 24 h as described in Materials and Methods. At 6 hpi and 24 hpi, protein extracts were analyzed by Western blotting using antibodies reactive to mGbp2 and actin. Densitometric analyses represent the ratio of intensity of the corresponding mGbp2 protein to actin expression per unit area, normalized over 6-h uninfected control and are represented as an arbitrary unit. Data are representative of three independent experiments. Values are means ± standard deviations (SD) (error bars). Statistical significance was measured, using two-tailed unpaired Student’s *t* test relative to the uninfected control in each time point, and indicated as follows: *, *P* < 0.05; **, *P* < 0.01; ***, *P* < 0.001.

10.1128/mBio.01464-20.1 FIG S1 Differential Irgb10 expression in L. donovani-infected macrophages and MEFs. (A) Peritoneal macrophages and MEFs, derived from C57BL/6 mice, were either unprimed or primed overnight with 200 U/ml of IFN-γ, and the protein extracts were analyzed by Western blotting using antibodies reactive to mGbp2 and actin. (B) Peritoneal macrophages and MEFs were either infected with L. donovani strains AG83 and DD8 or left uninfected. At 6 hpi and 24 hpi, protein extracts were analyzed by Western blotting using antibodies reactive to mIrgb10 and actin (the same blot was used as in Fig. 2). Densitometric analyses represent the ratio of intensity of the corresponding mIrgb10 protein to actin expression per unit area, normalized over 6-h uninfected control and are represented as an arbitrary unit. Data are representative of two independent experiments. Results are expressed as means ± SD (error bars). Statistical significance was measured, using two-tailed unpaired Student’s t test relative to the uninfected control in each time point, and indicated as follows: *, *P* < 0.05; **, *P* < 0.01. Download FIG S1, TIF file, 0.8 MB. Copyright © 2020 Haldar et al. 2020 Haldar et al. This content is distributed under the terms of the Creative Commons Attribution 4.0 International license.

### Differential expression of mGbp2 in L. donovani-infected macrophages and MEFs.

IFN-γ-inducible immunity-related GTPases (IRGs) and GBPs are important intracellular effectors against intracellular pathogens like Toxoplasma gondii (3, 4, 25, 26, 44). In response to infection with intracellular bacterial and protozoan pathogens, these IFN-γ-inducible GTPases, including GBPs, are transcriptionally upregulated. In previous studies, global gene expression analyses showed induction of mouse *Gbp1/2/3/6/7* expression in L. major-infected murine BMDMs (38) and increased expression of *Gbp1* and *Gbp5* in the skin, inguinal lymph nodes, spleen, and liver when mice were infected with L. major (39). Expanding these studies to L. donovani, we measured *Gbp* expression at 6 and 24 hpi in macrophages and MEFs infected with two different L. donovani strains (AG83 and DD8). Cell lysates were analyzed by Western blotting to assess mGbp2 protein expression. Infections with either AG83 or DD8 induced robust Gbp2 protein expression in both macrophages and MEFs (9- to 14-fold increase in macrophages and 5- to 8-fold increase in MEFs compared to uninfected controls at the indicated time postinfection) (Fig. 2A and B). We also monitored protein expression of mouse Irgb10, one of the effector IRGs, in murine cells during L. donovani infections and observed a 2.5- to 9-fold increase in Irgb10 expression in response to infections at 24 hpi but noticed no significant change in expression at early times of infection (Fig. S1B).

### Mouse Gbps do not efficiently target L. donovani-containing vacuoles in MEFs.

**FIG 3:**
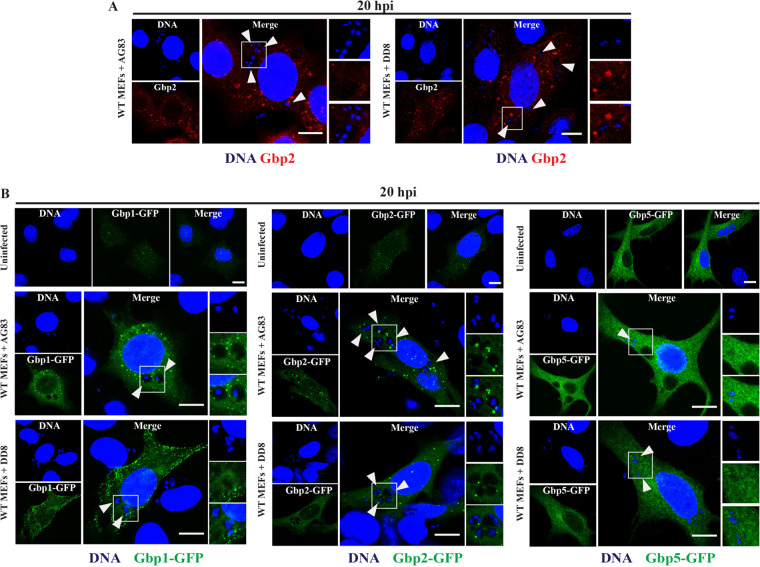
mGbps do not localize to the intracellular L. donovani-containing vacuoles (LCVs) in MEFs. (A) WT MEFs, primed overnight with 200 U/ml IFN-γ, were infected with L. donovani strain AG83 or DD8, and at 20 hpi, the infected cells were fixed and stained with rabbit anti-mGbp2 (red) and DNA (blue). (B) MEFs engineered to express mGbp1-GFP, mGbp2-GFP, and mGbp5-GFP were either infected with L. donovani strain AG83/DD8 or left uninfected, and at 20 hpi, the GFP-targeting LCVs were monitored. Arrowheads indicate LCVs. Representative confocal images are shown here. All bars = 10 μm.

Several mGbps translocate to the pathogen-containing vacuoles (PVs), disrupt PV integrity, and facilitate the destruction of pathogens. Previous reports showed that mGbp2 localizes to PVs containing Chlamydia trachomatis, Salmonella enterica serotype Typhimurium, Legionella pneumophila, and Toxoplasma gondii in murine fibroblasts, epithelial cells, macrophages, and spleen tissues (12, 45–54). Accordingly, we explored whether or not mGbp2 was able to target L. donovani-containing vacuoles (LCVs). To do so, we infected IFN-γ-primed cells with L. donovani strain AG83 or DD8 and checked both endogenous and ectopically expressed mGbp2 recruitment to LCVs. Unexpectedly, immunofluorescence microscopy showed that neither endogenous mGbp2 nor ectopically expressed mGbp2-green fluorescent protein (GFP) detectably decorated LCVs in MEFs or in macrophages (Fig. 3A and B and Fig. S2A). This lack of recruitment was observed at all time points tested and was not affected by the absence or presence of IFN-γ priming (Fig. S2A and B). We extended our observation to additional mGbp family members and failed to observe any LCV targeting of ectopically expressed mGbp1-GFP and mGbp5-GFP (Fig. 3B). Similar to what we observed for mGbp, we failed to detect Irgb10 localization to LCVs (Fig. S2C).

10.1128/mBio.01464-20.2 FIG S2 mGbp2 and Irgb10 do not localize to the intracellular L. donovani-containing vacuoles (LCVs) in murine cells. (A) Peritoneal macrophages, primed overnight with 200 U/ml IFN-γ, were infected with L. donovani strain AG83 or DD8, and at 4 hpi and 20 hpi, the infected cells were fixed and stained with rabbit anti-mGbp2 (red) and DNA (blue). (B) Unprimed WT MEFs were infected with L. donovani strain AG83 or DD8, and at 20 hpi, the infected cells were fixed and stained with rabbit anti-mGbp2 (red) and DNA (blue). (C) WT MEFs expressing mIrgb10-GFP were either left uninfected or infected with L. donovani strains AG83/DD8, and at 20 hpi, the GFP-targeting LCVs was monitored. Representative confocal images are shown here. Arrowheads indicate LCVs. All scale bars = 10 μm. Download FIG S2, TIF file, 1.2 MB. Copyright © 2020 Haldar et al. 2020 Haldar et al. This content is distributed under the terms of the Creative Commons Attribution 4.0 International license.

### Mouse Gbps reduce L. donovani burden in MEFs in an IFN-γ-independent manner.

**FIG 4:**
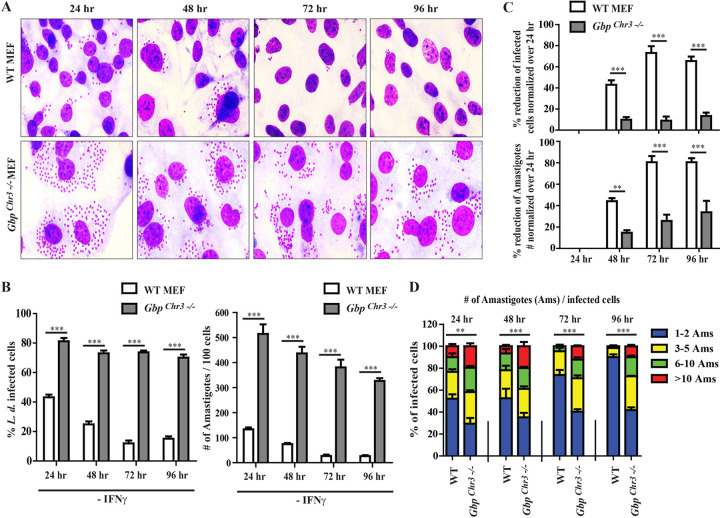
mGbps on chromosome 3 (*Gbp^chr3^*) promote cell-autonomous host defense against L. donovani infection in MEFs. WT and *Gbp^chr3−/−^* MEFs were infected with stationary-phase L. donovani strain AG83 as described in Materials and Methods. At 6 hpi, floated parasites were washed and incubated further for the indicated time points. At 24, 48, 72, and 96 hpi, cells were fixed with methanol and stained with Giemsa. (A) Representative microscopic images of unprimed infected cells at different time points are shown here. (B) By using light microscopy, the number of infected cells and the number of intracellular parasites at 24, 48, 72, and 96 hpi were assessed via the quantification of parasite-containing cells and number of amastigotes, respectively, as described in Materials and Methods. (C) Percent reduction of infected cells over time was calculated as [100 − (percentage of infected cells at the indicated time postinfection/percentage of infected cells at 24 hpi) × 100]. Similarly, percent reduction of amastigote numbers over time was calculated as [100 − (number of intracellular amastigotes per 100 cells at indicated time postinfection/number of intracellular amastigotes per 100 cells at 24 hpi) × 100]. Results are expressed as means ± SEM and are based on three independent experiments run in triplicate. (D) Parasite survival was determined by counting the number of parasites within infected MEFs at the specified time postinfection. Results are expressed as means ± SEM (*n* = 3). Statistical significance was analyzed by two-way ANOVA and indicated as follows: **, *P* < 0.01; ***, *P* < 0.001.

Up to this point, our data revealed that LCV membranes generally remained devoid of mGbps in L. donovani-infected MEFs. We considered two potential explanations for this unexpected finding. (i) L. donovani may interfere with mGbp function to escape from mGbp-mediated immunity in MEFs, as reported for several *Toxoplasma* strains as well as Chlamydia muridarum (54–56). Alternatively, (ii) mGbp-mediated host defense to L. donovani does not require mGbp translocation of LCVs. To distinguish between these two possibilities, we monitored L. donovani burden in wild-type and *Gbp^chr3−/−^* MEFs, which lack the chromosome 3 *Gbp* gene cluster encompassing *Gbp1*, *Gbp2*, *Gbp3*, *Gbp5*, and *Gbp7* (57). We primed *Gbp^chr3−/−^* MEFs and corresponding wild-type MEFs with IFN-γ overnight or left the cells untreated and subsequently infected these cells with L. donovani strain AG83 or DD8 for 6 h. At 6 h postinfection (hpi), cells were washed to remove extracellular parasites and parasite growth was continuously monitored at 24, 48, 72, and 96 hpi by counting the percentage of infected cells as well as the total number of parasites per 100 cells. We observed that both the percentage of AG83 infected cells and the total parasite burden in *Gbp^chr3−/−^* MEFs were significantly elevated relative to wild-type cells (∼2-fold and ∼5-fold, respectively) at 24 hpi (Fig. 4A and B). Next, we quantified the efficacy with which wild-type and *Gbp^chr3−/−^* MEFs inhibited parasite growth and survival from 24 to 96 hpi. We observed pronounced reductions in the percentages of infected cells (65.49% ± 4.24%) and the total parasite numbers (80.54 ± 3.78%) in wild-type MEFs during this time interval. The reduction in the percentage of infected cells (13.38% ± 3.24%) and total parasite burden (33.81% ± 10.61%) was less pronounced in unprimed (Fig. 4C and Fig. S3A) as well as IFN-γ-primed *Gbp^chr3−/−^* MEFs (Fig. S3B). Similar trends were also observed in MEFs infected with L. donovani strain DD8 (Fig. S3C). Notably, a higher number of individual *Gbp^chr3−/−^* than wild-type MEFs contained 10 or more parasites per cell at 48, 72, and 96 hpi (Fig. 4D), further underscoring the ability of mGbps to restrict L. donovani burden in a cell-autonomous manner.

10.1128/mBio.01464-20.3 FIG S3 mGbps on chromosome 3 (*Gbp^chr3^*), but not Irgms, promote cell-autonomous host defense against L. donovani infection in MEFs in an IFN-γ-independent manner. WT, *Gbp^chr3−/−^*, and *Irgm1/3^−/−^* MEFs, either unprimed or primed overnight with IFN-γ were infected with L. donovani strain AG83 (A and B) or DD8 (C) as described in the legend to Fig. 4. At 6 hpi, floated parasites were washed and incubated further for the indicated time points. At 24, 48, 72, and 96 hpi, cells were fixed with methanol and stained with Giemsa. (A) Representative microscopic images of unprimed infected *Irgm1/3^−/−^* MEFs at different time points are included in Fig. 4A and shown here. (B and C) By using light microscopy, the number of infected cells and the number of intracellular parasites at 24, 48, 72, and 96 hpi were assessed via the quantification of parasite-containing cells and the numbers of amastigotes, respectively, as described in Materials and Methods. The data for *Irgm1/3^−/−^* MEFs and IFN-γ-primed conditions are included in Fig. 4B and shown here. Results are expressed as means ± standard errors of means (SEM) and are based on three independent experiments run in triplicate. Statistical significance was analyzed by two-way ANOVA and indicated as follows: *, *P* < 0.05; **, *P* < 0.01; ***, *P* < 0.001; ns, not significant. Download FIG S3, TIF file, 1.6 MB. Copyright © 2020 Haldar et al. 2020 Haldar et al. This content is distributed under the terms of the Creative Commons Attribution 4.0 International license.

### Irgm1 and Irgm3 are not required for cell-autonomous immunity to L. donovani in MEFs.

We and others previously demonstrated that GBP-mediated innate immune recognition of bacterial and protozoan PVs is dependent on the immunity-related GTPase family M protein (IRGM) subset of IRG proteins and that mice lacking Irgm1 and Irgm3 expression are more susceptible to *Toxoplasma* and C. trachomatis infections in cell culture and *in vivo* (54, 58–64). To determine whether mGbp-mediated cell-autonomous host defense to *Leishmania* was similarly dependent on IRGM, we monitored L. donovani infectivity and burden in *Irgm1/3^−/−^* MEFs. Although parasitic burden was moderately diminished overall in *Irgm1/3^−/−^*MEFs possibly due to a defect in endocytosis, we found that wild-type and *Irgm1/3^−/−^*MEFs controlled L. donovani amastigote numbers in a comparable manner (Fig. S3A and B). Collectively, these data indicate that control of L. donovani burden by mGbps operates independent of IRGM proteins.

### Human GBP1 restricts L. donovani burden in human A549 cells independent of targeting LCVs.

**FIG 5:**
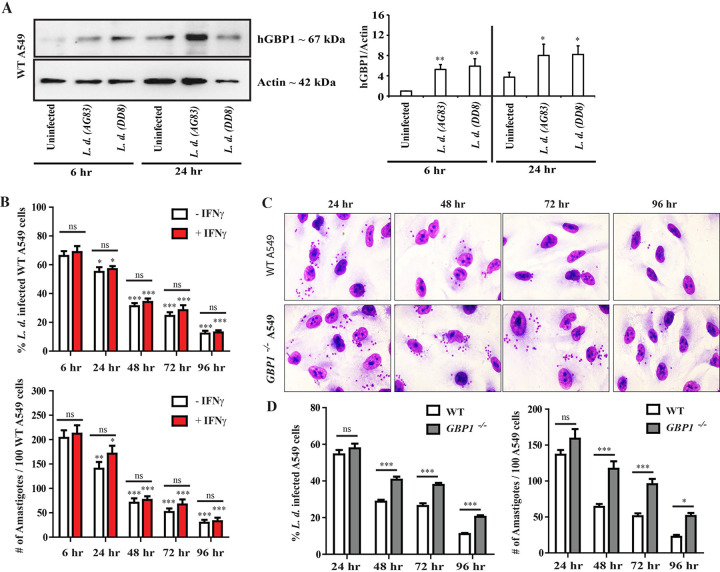
hGBP1 promotes cell-autonomous host defense against L. donovani infection in human epithelial A549 cells by an IFN-γ-independent manner. (A) WT A549 cells were either infected with L. donovani strains AG83 and DD8 for 6 h and 24 h or left uninfected. At 6 hpi and 24 hpi, protein extracts were analyzed by Western blotting using antibodies reactive to hGBP1 (Santa Cruz) and actin. Densitometric analyses represent the ratio of intensity of the corresponding hGBP1 protein to actin expression per unit area and are represented as an arbitrary unit. Data are representative of three independent experiments. Values are means ± SD (error bars). (B) WT A549 cells were either left unprimed or primed overnight with 200 U/ml of hIFN-γ and infected with L. donovani strain AG83 for the indicated time points. (C and D) WT and *hGBP1^−/−^* A549 cells were infected with strain AG83 for 24, 48, 72, and 96 h as described in Materials and Methods. At 6 hpi, floated parasites were washed and incubated further for the indicated time points. At the specified time postinfection, cells were fixed with methanol and stained with Giemsa. By using light microscopy, the number of infected cells and the number of intracellular parasites at 6, 24, 48, 72, and 96 hpi were assessed via the quantification of parasite-containing cells and number of amastigotes, respectively, as described in Materials and Methods. (C) Representative microscopic images of unprimed infected cells at different time points are shown here. (D) Results are expressed as means ± SEM and are based on three independent experiments run in triplicate. Statistical significance was analyzed by two-way ANOVA, between 6 hpi versus other indicated time postinfection or otherwise indicated by bars, and indicated as follows: *, *P* < 0.05; **, *P* < 0.01; ***, *P* < 0.001; ns, not significant.

**FIG 6:**
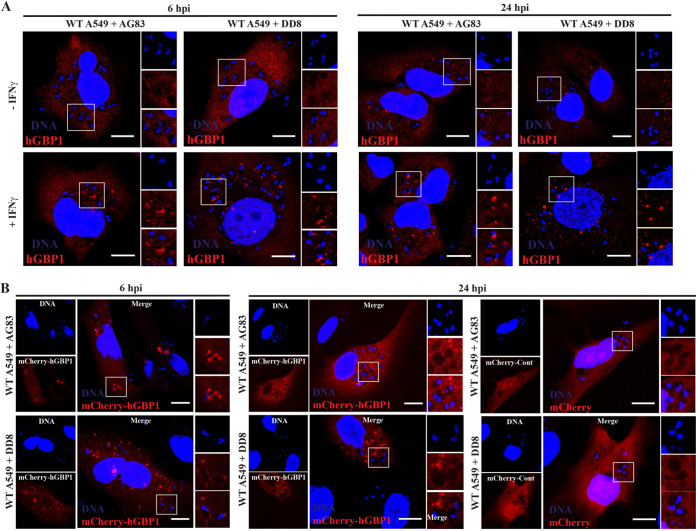
hGBP1 does not localize to the intracellular L. donovani-containing vacuoles (LCVs). (A) WT A549 cells, either unprimed or primed overnight with 200 U/ml IFN-γ, were infected with L. donovani strain AG83 or DD8, and at 6 hpi and 24 hpi, the infected cells were fixed and stained with anti-hGBP1 (red) and DNA (blue). (B) Immunofluorescent confocal images of LCVs at 6 hpi and 24 hpi in mCherry-control- and mCherry-hGBP1-expressing A549 cells, primed overnight with 200 U/ml IFN-γ, are shown here. All scale bars = 10 μm.

The *Gbp* gene cluster on mouse chromosome 3 is syntenic to the region of human chromosome 1 encompassing a set of seven annotated human *GBP* paralog (7). While the antimicrobial activities of GBPs so far have been studied more extensively in the mouse model, an increasing number of studies on hGBPs revealed comparable activities between mouse and human GBPs (3, 4, 8, 26). A previous report showed that the dendritic cells derived from healthy human blood exhibited increased expression of GBP1 during L. donovani infection (40). Expanding this observation to human nonphagocytic A549 cells, we measured GBP1 expression at 6 and 24 hpi in A549 cells infected with two different L. donovani strains (AG83 and DD8). We observed that infections with either AG83 or DD8 induced two- to six-fold higher GBP1 protein expression in A549 cells compared to uninfected controls at different time points tested (Fig. 5A). Another report demonstrated that hGBP1 restricted *Toxoplasma* replication independent of hGBP1 recruitment to the *Toxoplasma* PV in A549 cells (65), leading us to hypothesize that hGBP1 in A549 cells executes antiparasitic host defense program that is functionally related to the GBP-mediated anti-*Leishmania* defense we observed in MEFs. To test this hypothesis, we monitored L. donovani burden in IFN-γ-primed and unprimed A549 cells over the course of 96 h. In support of our hypothesis, we observed that parental A549 cells were able to kill L. donovani AG83 in an IFN-γ-independent manner (Fig. 5B) and that much of this restriction was dependent on hGBP1 at 48, 72, and 96 hpi (Fig. 5C and D). Next, we asked whether or not hGBP1 associates with LCVs in A549 cells. To do so, we monitored the subcellular localization of human GBP1 either by using anti-hGBP1 antibody or by ectopically expressing mCherry-labeled human GBP1 (mCherry-hGBP1) fusion protein in IFN-γ-primed and unprimed A549 cells following L. donovani infection. Neither endogenous hGBP1 nor mCherry-hGBP1 were detectable at LCVs at any of the time points tested (Fig. 6A and B). Collectively, these data suggested that hGBP1 promote parasite clearance from infected A549 cells in a process that does not require efficient targeting of hGBP1 to LCVs.

### Mouse Gbps and human GBP1 facilitate the recruitment of lysosomal markers LAMP and autophagy marker LC3 to the LCVs.

**FIG 7:**
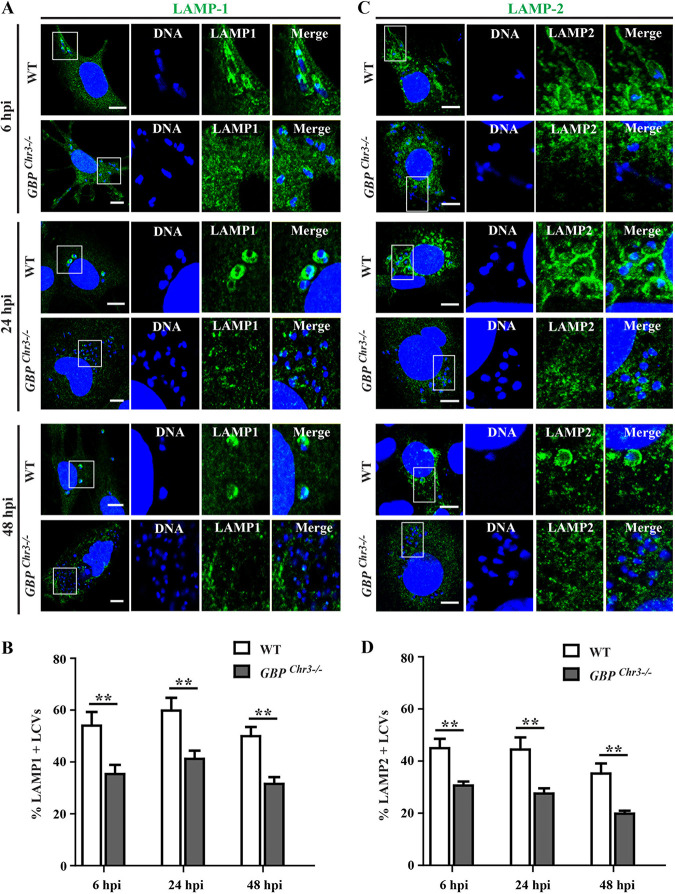
mGbps facilitate LAMP1 and LAMP2 recruitment to parasitophorous vacuoles harboring L. donovani in MEFs. WT and *Gbp^chr3−/−^* MEFs were infected with stationary-phase L. donovani promastigotes. At 6 hpi, 24 hpi, and 48 hpi, cells were stained for either rabbit anti-LAMP1 (green) (A) or LAMP2 (green) (C) and DNA (blue). Representative confocal images of LAMP1/LAMP2-positive vacuoles are shown. LAMP1-positive (B) or LAMP2-positive (D) *Leishmania*-containing vacuoles (LCVs) in MEFs was quantified as described in Materials and Methods. Results are expressed as means ± SEM and are based on three independent experiments run in duplicate. Statistical significance was analyzed by two-way ANOVA and indicated as follows: **, *P* < 0.01. Bars = 10 μm.

**FIG 8:**
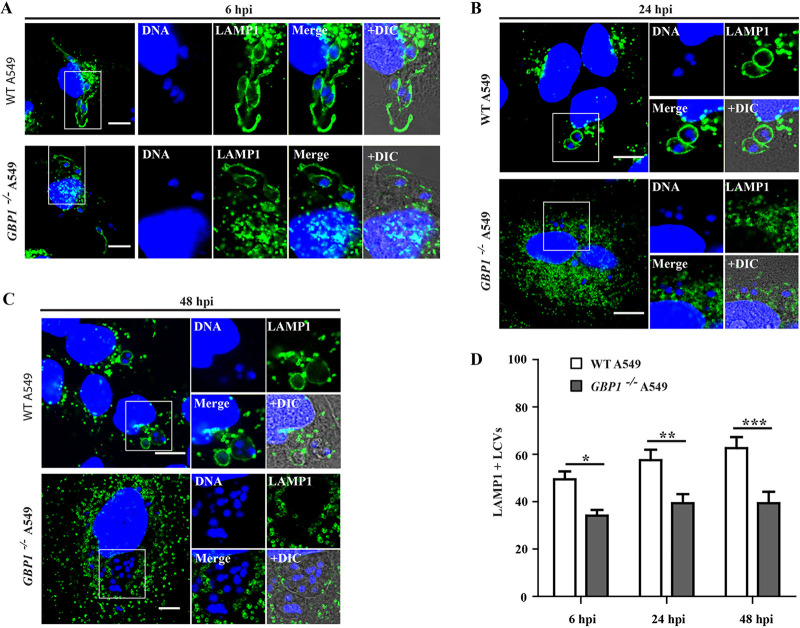
hGBP1 facilitates LAMP1 recruitment to parasitophorous vacuoles harboring L. donovani in A549 cells. WT and *GBP^−/−^* A549 cells were infected with stationary-phase AG83 promastigotes. At 6 hpi (A), 24 hpi (B), and 48 hpi (C), cells were stained for mouse anti-LAMP1 (green) and DNA (blue). Representative confocal images of LAMP1-positive vacuoles are shown. (D) LAMP1 recruitment with *Leishmania*-containing vacuoles (LCVs) in A549 cells was quantified as described in Materials and Methods. Results are expressed as means ± SEM and are based on three independent experiments run in duplicate. Statistical significance was analyzed by two-way ANOVA and indicated as follows: *, *P* < 0.05; **, *P* < 0.01; ***, *P* < 0.001. Bars = 10 μm.

Many intracellular pathogens evolved diverse strategies to block the fusion of PVs with degradative antimicrobial lysosomes (66–69). *Leishmania* spp. taken up by phagocytosis into macrophages block the rapid fusion of these *Leishmania-*containing vacuoles with lysosomes (70–72). In contrast to the mechanism by which *Leishmania* enters macrophages, it was recently reported that L. amazonensis actively invades MEFs via endocytosis, and further, that those endocytosed parasites acquire the lysosomal markers lysosome-associated membrane proteins (LAMPs), LAMP1 and LAMP2 (37). To investigate whether intracellular L. donovani also colocalizes with the endolysosomal system in MEFs, we infected wild-type (WT) MEFs with L. donovani for 6, 24, and 48 h and stained cells with anti-LAMP1 and anti-LAMP2 antibodies to assess the recruitment of LAMP1/LAMP2 to LCVs by fluorescence microscopy. We observed that about 50 to 70% of LCVs were LAMP1 positive and 35 to 55% of LCVs were LAMP2 positive at different time points tested (Fig. 7A to D). Notably, most of these LAMP-positive LCVs contained only one or two parasites, whereas LCVs containing multiple parasites were mostly devoid of LAMP decoration, thus suggesting that LAMP-positive LCVs are nonpermissive for parasite growth and survival (Fig. 7 and Fig. S4). Since we identified mGbps as executioners of anti-*Leishmania* host defense in MEFs (Fig. 4), we reasoned that mGbps could control LAMP acquisition by LCVs in MEFs. In support of this hypothesis, we found that the number of LAMP1^+^/LAMP2^+^ LCVs was significantly diminished in *Gbp^chr3−/−^* MEFs (Fig. 7A to D). Comparable to our observations in MEFs, we found that the number of LAMP1-positive (LAMP1^+^) LCVs were also significantly reduced in *GBP1^−/−^* A549 cells (35%, 39%, and 39% at 6 h, 24 h, and 48 h, respectively) compared to WT A549 cells (about 50%, 58%, and 63% at 6 h, 24 h, and 48 h, respectively) (Fig. 8A to D).

10.1128/mBio.01464-20.4 FIG S4 LAMP-1 and LAMP2 recruitment to parasitophorous vacuoles harboring L. donovani. WT MEFs (A and B) and WT A549 cells (C) were infected with stationary-phase AG83 promastigotes. At the indicated time points, cells were stained for either rabbit anti-LAMP1 (green [A] or red [C]) or LAMP2 (green [B]) and DNA (blue). Representative confocal images of LAMP1- or LAMP2-positive vacuoles are shown. White arrowheads indicate LAMP-positive vacuoles, and white arrows indicate LAMP-negative vacuoles. Scale bars = 10 μm. Download FIG S4, TIF file, 1.8 MB. Copyright © 2020 Haldar et al. 2020 Haldar et al. This content is distributed under the terms of the Creative Commons Attribution 4.0 International license.

**FIG 9:**
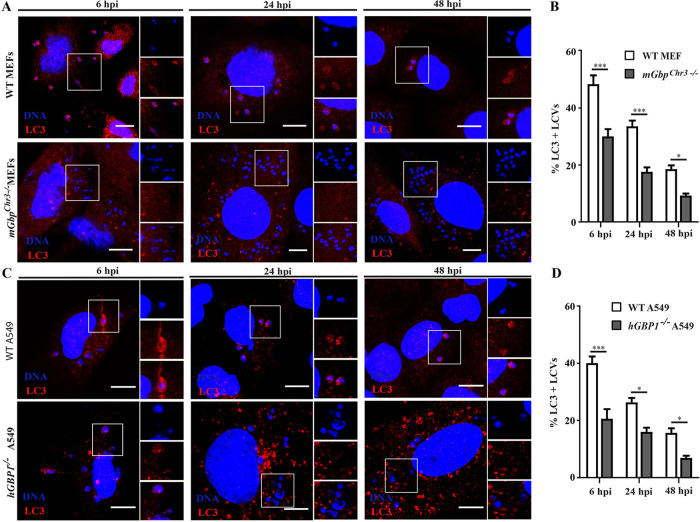
mGbps and hGBP1 facilitate LC3 recruitment to parasitophorous vacuoles harboring L. donovani in MEFs and A549 cells, respectively. WT and *mGbp^chr3−/−^* MEFs (A and B) or WT and *hGBP^−/−^* A549 cells (C and D) were infected with stationary-phase L. donovani promastigotes. At 6, 24, and 48 hpi, cells were stained for rabbit anti-LC3 (red) and DNA (blue). (A and C) Representative confocal images of LC3-positive vacuoles are shown. (B and D) LC3 recruitment to *Leishmania*-containing vacuoles (LCVs) in MEFs and A549 cells was quantified as described in Materials and Methods. Results are expressed as means ± SEM and are based on three independent experiments run in duplicate. Statistical significance was analyzed by two-way ANOVA and indicated as follows: *, *P* < 0.05; ***, *P* < 0.001. Bars = 10 μm.

**FIG 10:**
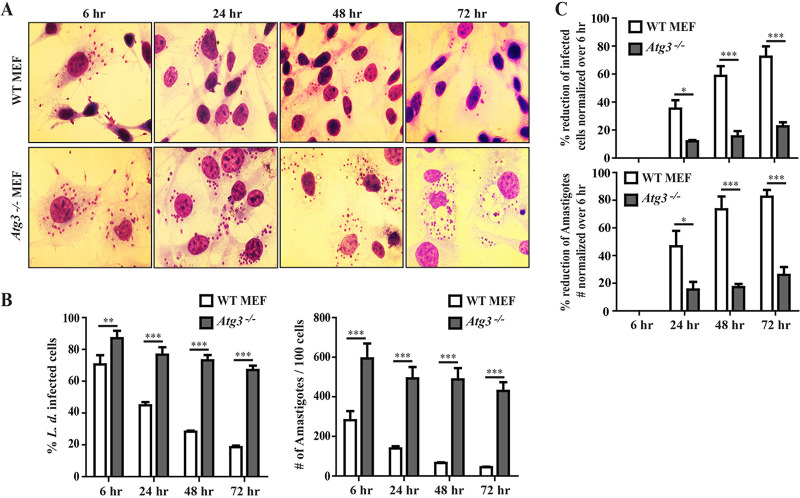
Mouse Atg3 promotes cell-autonomous host defense against L. donovani infection in MEFs. WT and *Atg3^−/−^* MEFs were infected with stationary-phase L. donovani strain DD8 as described in Materials and Methods. At 6 hpi, floated parasites were washed and incubated further for the indicated time points. At 6, 24, 48, and 72 hpi, cells were fixed with methanol and stained with Giemsa. (A) Representative microscopic images of unprimed infected cells at different time points are shown here. (B) By using light microscopy, the number of infected cells and the number of intracellular parasites at 6, 24, 48, and 72 hpi were assessed via the quantification of parasite-containing cells and number of amastigotes, respectively, as described in Materials and Methods. Results are expressed as means ± SEM and are based on two independent experiments run in triplicate. (C) Percent reduction of infected cells over time was calculated as [100 − (percentage of infected cells at the indicated time postinfection/percentage of infected cells at 6 hpi) × 100]. Similarly, percent reduction of amastigote numbers over time was calculated as [100 − (number of intracellular amastigotes per 100 cells at the indicated time postinfection/number of intracellular amastigotes per 100 cells at 6 hpi) × 100]. Results are expressed as means ± SEM. Significance was analyzed by two-way ANOVA and indicated as follows: *, *P* < 0.05; **, *P* < 0.01; ***, *P* < 0.001.

Our studies indicate that a GBP-dependent mechanism reroutes LCVs to a LAMP1^+^ compartment. Because GBP proteins were previously shown to regulate autophagy (73), we hypothesized that GBP-mediated delivery of L. donovani into a LAMP1^+^ compartment was dependent on an autophagy-related process. In support of this hypothesis, we found that the autophagosome marker light chain 3 (LC3) to colocalize with LCVs. The number of LC3-positive LCVs was significantly reduced in *Gbp^chr3−/−^* MEFs and *GBP1^−/−^* A549 cells compared to their wild-type counterparts at all time points tested (Fig. 9A to D), suggesting that GBPs promote the delivery of L. donovani into autolysosomes. In order to directly test the hypothesis that an autophagy-related process could drive the anti-*Leishmania* host response in fibroblasts, we monitored the ability of autophagy-deficient *Atg3*^−/−^ MEFs to contain L. donovani burden over time. We observed that compared to wild-type MEFs, a greater percentage of *Atg3*^−/−^ MEFs remained infected with L. donovani throughout the 72-h time course of the infection and that the number of L. donovani amastigotes per host cells was significantly higher in *Atg3*^−/−^ MEFs compared to wild-type cells (Fig. 10A to C). Collectively, these data indicate that mGbps encoded on chromosome 3 and hGBP1 restrict *Leishmania* growth in nonphagocytic cells by an autophagy-related antiparasitic pathway.

## DISCUSSION

Professional phagocytic cells are considered the primary host cell type in which *Leishmania* survives and replicates. During the last few decades, nearly all *Leishmania* research has therefore focused on understanding the mechanism by which *Leishmania* co-opts phagocytic cells for its propagation. However, nonphagocytic cell types have also been shown to endocytose *Leishmania in vitro* and *in vivo* (34–36, 74). Several studies demonstrated the uptake of promastigotes or amastigotes of L. donovani, L. mexicana *amazonensis*, and an *L*. *braziliensis*-like species by human and mouse fibroblasts *in vitro* (29, 35–37, 75, 76). Although these observations suggest a potentially important role for nonphagocytic cells in the pathogenesis of leishmaniasis, almost no efforts were made to understand how these nonphagocytic cells interact with the parasite. In this study, we demonstrate an important role of IFN-γ-inducible guanylate binding proteins (GBPs) in the host defense to L. donovani in nonphagocytic mouse and human cells, thus defining a novel cell-autonomous immune pathway that renders nonphagocytic cells a hostile niche that is nonpermissive for robust intracellular *Leishmania* growth.

Previous gene microarray analyses showed upregulation of mouse Gbp1/2/3/6/7 expression in L. major-infected macrophages *in vitro* (38) and elevated Gbp1 and Gbp5 expression in the skin, lymph nodes, spleen, and liver in L. major-infected mice *in vivo* (39). While these studies provided important insights into the induction of GBP expression in response to *Leishmania* infections, a potential functional role of GBPs in host defense to *Leishmania* was not investigated at the time. Our study shows that mGbps encoded on mouse chromosome 3 mediate clearance of L. donovani in MEFs in a process that appears to operate independent of GBP translocation to PVs. Additionally, we demonstrate that hGBP1 promotes the elimination of L. donovani from human A549 cells, again without any notable translocation of hGBP1 to the LCVs. These data indicate that a GBP-dependent host defense pathway limiting *Leishmania* replication inside nonphagocytic cells is conserved between the murine and human host.

GBPs control multiple intracellular host defense activities against viral, bacterial, and protozoan pathogens that include the lytic disruption of PVs, the production of reactive oxygen species, and the delivery of antimicrobial molecules to PVs and viral replication complexes (1, 3, 4, 25–27, 77, 78). GBP-mediated host defense programs directed at *Toxoplasma* in MEFs are dependent on functional interactions between GBPs and a second family of IFN-inducible dynamin-related GTPases, the IRGs (12, 54, 57). IRGs are divided into two categories based on their structures and specific activities: (i) the predominantly cytosolic GKS-IRGs and (ii) the predominantly membrane-bound IRGM proteins. GKS-IRGs are known to directly associate with PVs, whereas IRGM proteins guard self-organelles and help orchestrate the targeting of GKS-IRGs to non-self PVs (59–64). We showed previously that the IRGM proteins Irgm1 and Irgm3 are required for the efficient delivery of not only GKS-IRGs but also GBPs to *Toxoplasma* PVs in MEFs. MEFs lacking Irgm1/m3 expression fail to deposit either GBPs or GKS-IRGs on *Toxoplasma* PV, and accordingly, *Irgm1/m3^−/−^* MEFs lose their capacity to control *Toxoplasma* intracellular replication (54, 61, 64). Here, we show that Irgm1/m3-deficient MEFs control L. donovani infection at least as efficiently as wild-type MEFs, demonstrating that the GBP-dependent cell-autonomous immune program clearing L. donovani infections is IRGM independent and likely mechanistically distinct from the GBP- and IRGM-dependent cell-intrinsic host response that controls *Toxoplasma* infections in this cell type. This model is further supported by our observation that mGBPs and the GKS-IRG Irgb10, localized to the *Toxoplasma* PV during a productive host response, are absent from LCVs. A previous report showing increased L. donovani burden in Irgm1- and/or Irgm3-deficient mice at 14 days postinfection (79) is therefore unlikely the result of a defect in the cell-autonomous immune response of nonphagocytic but could be the consequence of either leukocytic defects associated with Irgm deficiencies or a potential role for Irgm proteins in cell-intrinsic immunity to *Leishmania* in phagocytes (63, 80).

As already alluded to, IRG-dependent GBP-mediated host defenses correlate with the translocation of IRGs and GBPs to parasitophorous vacuoles, (3, 4, 25–27, 73), whereas IRG-independent but GBP-executed host defenses, as described here, operate without any detectable translocation of IRGs/GBPs to LCVs. These observations not only suggest that IRG-dependent and -independent pathways are mechanistically distinct from each other but may also explain why the latter pathway operates efficiently in the absence of IFN-γ priming: as shown in this study, infection alone appears to induce little IRG but robust GBP expression at early times of postinfection, the latter presumably being sufficient to execute the IRG-independent antiparasitic pathway reported here.

*Leishmania* predominantly replicates inside professional phagocytes. Following phagocytosis, *Leishmania* inhibits phagosome maturation by preventing fusion of the *Leishmania*-containing phagosomes with lysosomes, as demonstrated by the impaired recruitment of the lysosomal marker LAMP1 to LCVs inside phagocytes (70, 72, 81, 82). In contrast to phagocytes, we demonstrate in this study that nonphagocytic MEFs as well as human A549 cells promote the maturation of LCVs into LAMP1/2^+^ compartments. We further demonstrate that the association of these lysosomal markers with LCVs is controlled by GBPs both in mouse and human cells. The detailed mechanism by which GBPs promote the recruitment of lysosomal marker to the LCVs needs to be explored. Since GBPs fail to localize to LCVs, it is unlikely that they directly alter LCV properties in a way that would promote their fusion with lysosomes or their capture within autolysosomes. Instead, it appears more likely that GBPs operate indirectly, for example by boosting autophagic flux synergizing with other cell-autonomous immune programs that mark LCVs for antimicrobial attack (73). In support of this model, we observed that the autophagy marker LC3 is enriched on LCVs in the presence of mGbp^chr3^ in MEFs or hGBP1 in A549 cells, respectively. Moreover, we demonstrate that MEFs lacking the autophagy-related gene *Atg3* are similarly defective for cell-autonomous immunity toward L. donovani as *Gbp^chr3−/−^* MEFs, supporting the hypothesis that GBPs promote the capture of L. donovani inside autolysosomes. Future work will interrogate the cross talk between GBPs and autophagy-related host responses during *Leishmania* infection in nonphagocytic cells.

Whether the GBP-dependent anti-*Leishmania* response described here also operates in L. donovani-infected phagocytic cells also remains to be investigated. Some predictions can be made based on previous work suggesting that autophagy and related pathways play a limited role in anti-*Leishmania* cell-intrinsic immunity in phagocytes. Rather, it has been proposed that *Leishmania* may be able to co-opt the host’s autophagic machinery to promote parasitic replication inside macrophages (83–86). We demonstrate here that in contrast to phagocytes, GBP-dependent autophagy-related host responses play a central role in executing anti-*Leishmania* defense in fibroblasts. We would therefore predict that GBPs play a limited role in protecting macrophages from *Leishmania* infections. Instead, anti-*Leishmania* host defense in phagocytes is largely dependent on the production of reactive oxygen and nitrogen species upon parasite infection by phagocytosis (87–91).

In conclusion, we demonstrated in this study that, unlike *Toxoplasma* PVs, which are targeted directly by mouse Gbps leading to PV lysis, LCVs are devoid of mGbps or hGBP1 but are nonetheless delivered into autolysosomal compartments in a GBP-dependent manner. Further investigations are needed to delineate the molecular mechanism by which GBPs controls the delivery of *Leishmania* into a lysosomal compartment and whether this host defense program is also activated against other *Leishmania* species, including L. major. An improved understanding of this novel GBP-mediated host defense to *Leishmania* may provide a path toward the development of alternative therapeutic interventions to treat *Leishmania* infections.

## MATERIALS AND METHODS

### Host cell cultures.

Mouse embryonic fibroblasts (MEFs) derived from wild-type (WT), *Gbp^chr3−/−^*, *Irgm1/3^−/−^*, and *Atg3^−/−^* mice were previously described (51, 57, 64). MEFs and adenocarcinomic human alveolar basal epithelial A549 cells were cultured in Dulbecco’s modified Eagle’s medium (DMEM) with glutamine and supplemented with 5% heat-inactivated fetal bovine serum (FBS) (Gibco by Life Technologies) and cultured at 37°C in 5% CO_2_. A *GBP1*-deficient A549 cell line was previously reported (92). Where appropriate, cells were stimulated overnight by the addition of mouse or human gamma interferon (IFN-γ) (Millipore) to growth media.

### Animal and parasite strains.

Adult (12- to 14-week-old) C57BL/6 mice, reared in institute facilities, were used for experimental purposes with prior approval of the animal ethics committee of CSIR-Central Drug Research Institute, Lucknow, India. Two World Health Organization reference strains of Leishmania donovani, AG83 (MHOM/IN/83/AG83) and DD8 (MHOM/IN/80/DD8) (ATCC), originally isolated from Indian kala-azar patients, were maintained in golden hamsters as previously described (93, 94). Promastigotes obtained after transforming amastigotes from infected spleen were maintained in DMEM (Sigma) supplemented with HEPES, 10% FBS, and penicillin-streptomycin at 24°C.

### Isolation of peritoneal exudate cells.

Peritoneal exudate cells (PECs) were isolated as previously described (95). Briefly, autoclaved 4% starch suspension was administered intraperitoneally (i.p.) in adult C57BL/6 mice. Then the PECs were harvested 48 h after intraperitoneal elicitation with 4% starch and were recovered by flushing the peritoneal cavity with prechilled RPMI 1640 medium using a 22-gauge needle. The cells were then washed and resuspended in complete RPMI 1640 medium (supplemented with 10% FBS and penicillin-streptomycin) and adhered on petri plates or on coverslips as required for 4 to 6 h. Nonadherent cells, if any, were removed, and adherent PECs were incubated overnight in complete RPMI 1640 medium at 37°C before giving any treatment or infection.

### Infection of cells with L. donovani and enumeration of intracellular parasite number.

For immunocytochemistry and light microscopy, infection of MEFs or A549 cells with L. donovani was performed as follows. A 500-μl aliquot of MEFs/A549 single-cell suspension (1 × 10^5^ cells/ml) was added to 12-mm-diameter round coverslips in 24-well plate for the adherence of MEFs/A549 cells. Three technical replicates for each experimental condition per cell type were made unless stated otherwise. After 6 h of incubation at 37°C in 5% CO_2_, the cells were either treated with 200 U/ml of mouse/human recombinant IFN-γ or left untreated overnight in the CO_2_ incubator. The next morning, 400 μl of stationary-phase promastigotes (5 × 10^6^/ml) of either strain AG83 or DD8 were inoculated into the indicated wells and incubated for 4 to 6 h at 37°C. The cover slips were then washed with DMEM medium to remove floated parasites, 1 ml of complete medium containing 10% FBS was added to each cover slip, and incubated further at 37°C in 5% CO_2_ up to 24 h or 48 h or as indicated. After the indicated time points, the coverslips were washed with phosphate-buffered saline (PBS) of pH 7.2, dried, and fixed with high-performance liquid chromatography (HPLC)-grade methanol. The cells were then stained with Giemsa (Sigma). For each experimental condition and cell type, at least 10 randomly selected fields were imaged per cover slip and the numbers of infected cells and intracellular parasites were assessed as described elsewhere (92). Briefly, the percent infected cells = [(total number of infected cells in at least 10 fields per coverslip/total number of cells) × 100] and the number of amastigotes/100 cells = [(total number of amastigotes in 10 fields per coverslip/total number of cells) × 100]. The images were acquired on a Nikon Eclipse Ts2 inverted light microscope using a 60× objective. Tests were performed on different days in order to see the variation, if any.

For immunoblotting, cells were either primed overnight with mouse/human IFN-γ or left unprimed in 12-well plates. The next day, the cells were infected with L. donovani strains with a multiplicity of infection (MOI) of 1:25. At 6 hpi, the cells were washed with PBS to remove free parasites and kept for the indicated time periods at 37°C in 5% CO_2_. Then the cells were harvested, and protein samples from whole-cell lysates were prepared using cell lysis buffer containing NP-40 and protease inhibitor cocktail (Thermo-Fisher Scientific).

### Immunocytochemistry.

Immunocytochemistry was performed essentially as described previously (64). The cells were infected as stated above for the indicated time points. The cells were then washed thrice with PBS (pH 7.2) and fixed either with methanol for 3 min or with 4% paraformaldehyde for 20 min at room temperature. In all experiments involving paraformaldehyde fixation, fixed cells were permeabilized/blocked with 0.05% (wt/vol) saponin and 2% (wt/vol) bovine serum albumin (BSA) in PBS for 30 min at room temperature. Cells were stained with the indicated primary antibodies, followed by Alexa Fluor-conjugated secondary antibodies (Molecular Probes/Invitrogen). Nucleic DNA and parasitic DNA were stained with 4′,6′-diamidino-2-phenylindole (DAPI). Stained cells were washed with PBS, mounted on microscope slides with Mowiol (Sigma), and allowed to cure overnight. Cells were then imaged using a LICA (TCS-SP8) inverted confocal microscope. Colocalization of proteins with L. donovani-containing vacuoles (LCVs) was quantified in at least three independent experiments. To determine the frequency with which GBPs, Irgb10, LAMP1, LAMP2, and LC3 colocalize with LCVs, at least 50 to 100 LCVs were assessed for each experimental condition and cell type for every biological replicate. Differential interference contrast images were used to identify extracellular L. donovani.

### Antibodies and immunoblotting.

The primary antibodies used for immunocytochemistry were as follows: anti-Irgb10 rabbit polyclonal antiserum (64) at 1:1,000, anti-Gbp2 rabbit polyclonal (54, 64) at 1:500, anti-LAMP1 mouse monoclonal (Santa Cruz Biotechnology) at 1:250, anti-LAMP1 rabbit polyclonal (Gbiosciences) at 1:250, anti-LAMP2 rabbit polyclonal (Invitrogen) at 1:250, and anti-LC3 rabbit polyclonal (MBL International) at 1:250. Protein samples from whole-cell lysates were analyzed by sodium dodecyl sulfate-polyacrylamide gel electrophoresis (SDS-PAGE) and Western blotting. The blots were probed with primary antibodies specific for the following: anti-Gbp2 rabbit polyclonal at 1:1,000, anti-Irgb10 rabbit polyclonal antiserum at 1:1,000, anti-hGBP1 rat monoclonal at 1:1,000 (Santa Cruz Biotechnology), and anti-β-actin mouse monoclonal (Sigma) at 1:5000. Binding of secondary horseradish peroxidase (HRP)-labeled goat anti-rabbit IgG at 1:2,000 or goat anti-rat IgG at 1:2,000 or goat anti-mouse IgG antibodies at 1:5,000 (GeNei) was analyzed using Clarity Western ECL (Bio-Rad) or Westar Supernova chemiluminescent substrate (Cyanagen).

### Cell transfection.

MEFs and A549 cells were transfected using Lipofectamine LTX (Invitrogen) following the manufacturer’s instructions. Green fluorescent protein (GFP)-tagged expression constructs of mouse Gbp1 (mGbp1), mGbp2, mGbp5, and mIrgb10 have been previously described (46, 64). To monitor the subcellular localization of human GBP1 (hGBP1), cells were transfected with a previously described mCherry-hGBP1 expression construct (92).

### Statistical analysis.

Statistical analyses were performed using GraphPad Prism version 5.00 software and Excel. Statistical differences between the different cell types and between the different conditions within one group were determined using two-way analysis of variance (ANOVA) with Bonferroni *posthoc* comparisons. To analyze differences between two sets of independent data points, unpaired two-tailed *t* test was used. Tests were considered statistically significant if *P* < 0.05.
